# Manipulation of GameXPeptide synthetase gene expression by a promoter exchange alters the virulence of an entomopathogenic bacterium, *Photorhabdus temperata temperata*, by modulating insect immune responses

**DOI:** 10.3389/fmicb.2023.1271764

**Published:** 2023-12-18

**Authors:** Gahyeon Jin, Md Tafim Hossain Hrithik, Dong-Hee Lee, Il-Hwan Kim, Ji-Seon Jung, Helge B. Bode, Yonggyun Kim

**Affiliations:** ^1^Department of Plant Medicals, Andong National University, Andong, Republic of Korea; ^2^Industry Academy Cooperation Foundation, Andong National University, Andong, Republic of Korea; ^3^Korea Research Institute of Standards and Science, Daejeon, Republic of Korea; ^4^Department of Natural Products in Organismic Interactions, Max Planck Institute for Terrestrial Microbiology, Marburg, Germany; ^5^Molecular Biotechnology, Department of Biosciences, Goethe Universität Frankfurt, Frankfurt, Germany; ^6^Center for Synthetic Microbiology (SYNMIKRO), Phillips Universität Marburg, Marburg, Germany; ^7^Department of Chemistry, Phillips Universität Marburg, Marburg, Germany; ^8^Senckenberg Gesellschaft für Naturforschung, Frankfurt, Germany

**Keywords:** *Photorhabdus temperata temperata*, GXPs, NRPS, insect, immunity, virulence

## Abstract

An entomopathogenic bacterium, *Photorhabdus temperata* subsp. *temperata*, is mutualistic to its host nematode, *Heterorhabditis megidis*. The infective juvenile nematodes enter target insects through natural openings and release the symbiotic bacteria into the insect hemocoel. The released bacteria suppress the insect immune responses and cause septicemia through their secondary metabolites. GameXPeptide (GXP) is one of the common secondary metabolites of most *Photorhabdus* species and is produced by the catalytic activity of a specific non-ribosomal peptide synthetase called GxpS encoded by the *gxpS* gene. This study confirmed *gxpS* to be encoded in the *P. temperata temperata* genome and analyzed its expression during bacterial growth. LC-MS/MS analysis of the bacterial culture broth contained at least four different GXPs (GXP-A to GXP-D), in which GXP-A was the most abundant. To investigate GXP synthesis following *gxpS* expression, the *gxpS* promoter of *P. temperata temperata* was replaced with an inducible arabinose promoter by homologous recombination. The *gxpS* transcript levels in the mutant were altered by the addition of l-arabinose. Without the inducer, the *gxpS* transcript level was significantly lower compared to the wild type and produced significantly lower amounts of the four GXPs. The addition of the inducer to the mutant significantly increased *gxpS* expression and produced significantly higher levels of the four GXPs compared to the wild type. The metabolite extracts obtained from wild-type and mutant bacteria showed differential immunosuppressive activities according to their GXP contents against the cellular and humoral immune responses of a lepidopteran insect, *Spodoptera exigua*. Interestingly, the *gxpS*-mutant bacteria showed less insecticidal activity compared to the wild type, whereas the addition of GXP to the mutant significantly restored insecticidal activity. These results suggest that the *gxpS* gene encoded in *P. temperata temperata* is responsible for the production of at least four different GXPs, which play crucial roles in bacterial virulence.

## 1 Introduction

Two pathogenic bacterial genera, *Photorhabdus* and *Xenorhabdus*, are mutualistic to the entomopathogenic nematodes, *Heterorhabditis* and *Steinernema*, respectively (Boemare, 2002). Although the bacteria independently originated, they share a common lifestyle, probably due to convergent evolution (Chaston et al., 2011). Infective juvenile (IJ) nematodes enter target insects through natural openings such as the mouth, anus, or spiracle and finally infect the insect hemocoel. Then, they release the symbiotic bacteria from their intestine into the insect hemocoel. The released bacteria are then changed into the pathogenic form from the mutualistic form and suppress insect immune responses to protect the host nematode and the bacteria themselves with their secondary metabolites (Neubacher et al., 2020). The life form change is caused by a promoter inversion near to genes associated with the nematode and bacterial adhesion (Somvanshi et al., 2012). Under immunosuppressive conditions, the bacteria grow and cause fatal septicemia to kill the insect. In the cadaver, the nematodes proliferate and produce the next IJs by re-association of the bacteria and the host nematodes to infect other target insects (Stock, 2019). In this pathogenic life form, the secondary metabolites produced by the bacteria also inhibit other microbial infections in the insect cadaver to maintain the monoxenic conditions (Yimthin et al., 2021). Thus, the secondary metabolites synthesized by the bacteria play crucial roles in nematode-bacterial mutualism.

Secondary metabolites include amino acid derivatives, peptides, polyketides, and/or hybrid natural products that are synthesized using polyketide synthetase, non-ribosomal peptide synthetase (NRPS), or other enzymes (Cimen et al., 2022). A large number of genes required for secondary metabolite production are encoded in bacterial genomes (Shi et al., 2022). GameXPeptides (GXPs) are cyclopentapeptides with an initial identification of four GXPs (GXP-A to GXP-D) from a bacterial culture broth of *Photorhabdus luminescens* TTO1 via a combination of labeling experiments with mass spectrometry (Bode et al., 2012). Later, additional four GXPs (E–H) were identified from the bacteria with different bacterial culture conditions by providing precursor compounds to the culture medium (Nollmann et al., 2015).

GXPs are commonly produced from *Xenorhabdus* and *Photorhabdus* (Tobias et al., 2017), and most secondary metabolites are species- or genus specifically found in the bacteria (Shi and Bode, 2018). This universal production in both genera suggests that GXPs may play crucial and common roles in the pathogenic cycle of nematode-bacterial mutualism. Shi et al. (2022) suggested that GXP-A has inhibitory activity against insect immunity because it inhibits hemocyte-spreading behavior and a cellular immune response measured by nodule formation. This immunosuppressive activity of GXP-A was applied to enhance the insecticidal activity of a commercial biopesticide, *Bacillus thuringiensis*, against a lepidopteran insect, *Spodoptera exigua* because insect immunity is one of the infection barriers to bacterial pathogenicity (Hrithik et al., 2022). GXP is produced by the catalytic activity of an NRPS called GXP synthetase (*gxpS*) because the heterologous expression of *gxpS* in *Escherichia coli* produces GXPs (Nollmann et al., 2015). However, it was not clear whether *gxpS* expression might be functionally related to bacterial virulence against insects.

An entomopathogenic bacterium, *Photorhabdus temperata temperata* ANU101, was isolated from *Heterorhabditis megidis* (Kang et al., 2004). Its bacterial culture broth was potent in inhibiting insect immune responses (Seo et al., 2012), suggesting the presence of GXP compounds. This study focused on bacterial virulence in relation to *gxpS* expression. To test the functional relationship between *gxpS* expression and bacterial virulence, this study used a mutagenesis strategy through promoter exchange of the *gxpS* gene with an inducible promoter according to the method described by Bode et al. (2015).

## 2 Materials and methods

### 2.1 Bacterial culture

Four different species of *Photorhabdus* were obtained from the Korean Agricultural Collection (KACC; Rural Development Administration, Jeonju, Republic of Korea) with accession numbers KACC91042 for *P. temperata temperata* (Ptt), KACC11928 for *P. luminescens kayaii* (Plk), KACC12284 for *P. temperate thracensis* (Pt), and KACC12282 for *P. luminescens akhurstii* (Pla). *Xenorhabdus hominickii* (Xh; Park et al., 2017) and *Xenorhabdus nematophila* (Xn; Park and Kim, 2000) were obtained from our frozen culture stock. These bacteria were cultured in tryptic soy broth (TSB, Difco, Sparks, MD, United States) for 72 h at 28°C in a shaking incubator at 180 rpm.

### 2.2 Insect rearing

Larvae of *S. exigua* were collected from onion fields in Andong, South Korea and fed with an artificial diet (Goh et al., 1991) at 27 ± 1°C. Under these conditions, they underwent five larval instars (L1–L5). A sugar solution (10%) was used for feeding adults.

### 2.3 Chemicals

Four GXPs were synthesized and prepared by AnyGen Co., Ltd. (Gwangju, Republic of Korea). Their sequences were cyclo[D-Leu/L-Leu/D-Val/L-Leu/D-Phe] for GXP-A, cyclo[D-Leu/L-Leu/D-Leu/L-Leu/D-Phe] for GXP-B, cyclo[D-Leu/L-Leu/D-Val/L-Leu/D-Leu] for GXP-C, and cyclo[D-Leu/L-Leu/D-Leu/L-Leu/D-Leu] for GXP-D. In addition, a stable isotope-labeled cyclic pentapeptide of GXP-A was synthesized as cyclo[D-Leu/L-Leu*/D-Val/L-Leu/D-Phe] (Leu*: ^13^C6, ^15^N) for GXP-A*. The purities of the synthetic peptides were over 97% based on the quality control using MALDI-TOF and LC-UV analyses by the manufacturer (Supplementary Figure S1). The peptides were prepared with dimethylsulfoxide (DMSO) at a concentration of 1 mM. The stock solutions were stored in the freezer and diluted with DMSO to prepare working solutions. Anticoagulant buffer (ACB) was prepared with 186 mM of NaCl, 17 mM of Na_2_-EDTA, and 41 mM of citric acid. Its pH was then adjusted to 4.5 with HCl. 4′,6-Diamidine-2′-phenylindole dihydrochloride (DAPI) and MTT (3-[4,5-dimethylthiazole-2-yl]-2,5-diphenyl tetrazolium bromide) were purchased from Sigma-Aldrich (Seoul, Republic of Korea). Alexa Fluor 488, as a dye of fluorescein isothiocyanate (FITC), was purchased from Thermo Fisher Scientific (Waltham, MA, United States). Phosphate-buffered saline (PBS) was prepared with 100 mM of phosphate and adjusted to a pH of 7.4 with 1 N NaOH.

### 2.4 Prediction of *gxpS* gene in the genome of *Photorhabdus temperata temperata*

The whole genome sequence of *P. temperata temperata* Meg1 was retrieved from GenBank (accession number: GCA_000722995.1). Ten NRPS genes were predicted from the genome using NCBI gene annotation. Functional modules for each *NRPS* and resulting products were predicted using antiSMASH bacterial version software.^1^ Based on the predicted products of the NRPS genes, a pentapeptide-producing NRPS gene was predicted as the *gxpS* gene of *P. temperata temperata*. To support the prediction, a phylogenetic analysis was performed with *gxpS* orthologs from four other bacterial species: *P. luminescens laumondii*, *P. luminescens akhurstii*, *Photorhabdus thracensis*, and Ptt, with accession numbers of AXG48275.1, QXF34648.1, AKH65701.1, and JGVH01000001.1, respectively. The phylogeny analysis was performed by MEGA6 program.^2^ Bootstrap values were obtained with 1,000 repetitions to support branching and clustering. A physical map of the annotated 10 *NRPS* loci was constructed by their alignment to a full genome of *P. luminescens laumondii* TT01 (GenBank accession number: NC_005126) with the BlastN searching machine of NCBI^3^ (Supplementary Table S1).

### 2.5 RNA extraction and cDNA preparation

The cultured bacteria were centrifuged to obtain cell pellets, to which 1 mL of TRIzol reagent (Invitrogen, Carlsbad, CA, United States) was added. The pellets were then subjected to the procedure described in the manufacturer’s manual. Each extracted RNA was resuspended in 50 μL of diethyl pyrocarbonate, and its concentration was determined using a spectrophotometer (NanoDrop, Thermo Fisher Scientific). The RNA purity was confirmed by the absorbance ratio (260/280 nm) greater than 1.8. The cDNA synthesis was carried out using 400 ng of the purified RNA and an RT PreMix (iNtRON Biotechnology, Seoul, Republic of Korea) containing random primers. No genomic DNA contamination in the purified RNA was confirmed by RT-PCR (see below) using RNA extract as template rather than cDNA.

### 2.6 RT-PCR and RT-qPCR

For RT-PCR, the cDNAs were amplified in specific target genes using Taq polymerase (GeneAll Biotechnology Co., Ltd., Seoul, Republic of Korea) with gene-specific primers (Supplementary Table S2). The PCR reaction was initiated with an initial denaturation at 95°C for 5 min. The subsequent 35 amplification cycles were performed at 95°C for 1 min, 53–55°C for 1 min, and 72°C for 1 min. The PCR reaction was finalized with an additional extension step at 72°C for 10 min. The PCR product was analyzed by 1% agarose gel electrophoresis to determine the presence of the amplified product. For RT-qPCR, the Power SYBR Green PCR Master Mix (Toyobo, Osaka, Japan) was used with gene-specific primers under the guidelines of Bustin et al. (2009). Quantitative analysis was performed using the comparative CT method (Livak and Schmittgen, 2001). All experiments were independently replicated three times.

### 2.7 Expression analysis of *gxpS* in TSB and insect

RNA was extracted at various time points for each bacterial culture to measure the expression level of *gxpS* during the bacterial growth. Wild-type *P. temperate temperata* stock was streaked on a TSB plate, and a single colony was cultured in 10 mL TSB medium at 180 rpm and 28°C for 18–20 h. The culture broth was then transferred to 1 L of TSB medium and incubated at 180 rpm and 28°C. For each of the designated time points (0, 12, 24, 36, 48, 60, and 72 h), 5 mL of bacterial culture was obtained for RNA extraction. Additionally, 1 mL of bacterial culture was used to measure the optical density (OD) at 600 nm at each time point. Each time point was replicated with different bacterial culture flasks.

To monitor the bacterial growth and *gxpS* expression in *S. exigua*, 2 μL (2 × 10^6^ colony-forming unit (CFU)/mL) of freshly cultured bacteria was injected into the hemocoel of each fifth instar (L5) larva of *S. exigua* using a microsyringe (Hamilton, Reno, NE, United States). After incubation for 2, 4, 6, 12, and 18 h at room temperature, hemolymph was collected. The collected 50 μL of hemolymph was spread onto TSB plates. After 18 h culture at 28°C, the number of colonies was counted. In addition, total RNAs were extracted from the larvae injected with the same amount of bacteria used for the bacterial growth analysis. An experimental unit for the RNA extraction was a whole body at each time point. This experiment was independently repeated three times using different larvae. Extracted RNAs were used for RT-qPCR as described above.

### 2.8 Extraction of secondary metabolites from bacterial culture broth

Test bacteria were cultured in 1 L of TSB for 72 h at 28°C with a shaking speed of 180 rpm. The bacterial culture broth was centrifuged at 12,500× *g* for 20 min at 4°C, and the resulting supernatant was mixed with 1 L of ethyl acetate. Using a separate funnel, the organic phase was collected. The aqueous phase was used two times to extract the organic phase, as described above. The combined 3 L extract of the organic phase was dried using a rotary evaporator (N-1110 Eyela, Tokyo, Japan) at 30°C. The resulting dried pellet that contained the metabolites was weighed and resuspended with DMSO to a concentration of 100 ppm.

### 2.9 Liquid chromatography-tandem mass spectrometry (LC-MS/MS) analysis of GXP compounds

The organic extract, as described above, was mixed with an internal standard, GXP-A*, and dissolved in DMSO to a final concentration of 5 μM. The samples were filtered through a disposable membrane filter unit (PVDF, 0.2 μm pore size, Thermo Fisher Scientific), followed by 1:1 dilution with methanol prior to LC-MS analysis. For the calibration curves, six-point calibration solutions of four GXP mixtures were prepared from 0 to 100 μM with DMSO. The LC-MS/MS analysis was performed using a Prominence 20AD series HPLC (Shimadzu, Japan) coupled with a 5600+ TripleTOF electrospray ionization triple quadrupole-time of flight mass spectrometer (AB Sciex, Framingham, MA, United States). For determination of the GXPs, analytes were injected into a KINETEX F5 column (2.6 μm, 100 × 3 mm i.d., Phenomenex, Torrance, CA, United States) with a binary gradient consisting of 20 mM ammonium formate and 80/20 of acetonitrile/20 mM ammonium formate for the mobile phases A and B, respectively, both with 0.1% formic acid. Separation was obtained at a flow rate of 400 μL/min with a linear gradient elution from 95:5 (A:B) to 20:80 (A:B) for 7 min, followed by a 4 min re-equilibration to the initial condition. The injection volume was 2 μL, and the ion transitions (*m*/*z*) were as follows: GXP-A (586.4 > 473.3), GXP-B (600.4 > 487.3), GXP-C (552.4 > 439.3), GXP-D (566.4 > 453.2), and GXP-A* (593.30 > 480.3). The source voltage and temperature were 5.5 kV and 500°C, respectively. Other ionization and fragmentation parameters were carefully optimized by monitoring the MS signal prior to sample analysis.

### 2.10 *gxpS* mutagenesis by a promoter exchange

The first 600 bp of the *gxpS* open reading frame (ORF) of *P. temperata temperata* was amplified with a forward primer (CATATGATGAAAGACAGTATTACCAG), which contained an Nde I restriction site (underlined) along with a start codon, and the reverse primer (CTGCAG CATGATATACGCCGGCCCGG), with a Pst I restriction site (underlined). The resulting PCR product was cloned into a pCEP plasmid (Bode et al., 2015) and transformed using *E. coli* S17. For conjugation using *E. coli* S17 as a donor to *P. temperata temperata*, both bacterial cells were grown in Luria–Bertani (LB) broth until reaching an optical density of 0.6–0.7 at 600 nm. Transformed cells (20 uL) of *E. coli* S17 were co-cultured with *P. tempera temperata* (60 uL) and spread onto an LB agar plate followed by incubating at 30°C. The cultured bacterial colonies exhibiting a red color (characteristic of *P. temperata temperate* colonies) were streaked onto selective LB agar containing kanamycin and incubated at 30°C for 2 days. Single colonies were analyzed by PCR with specific primers for the mutant *P. temperata temperata* (Supplementary Table S2). Each end of the resulting 5,823 bp product was sequenced to confirm the mutant (Supplementary Figure S2).

### 2.11 Induction of *gxpS* expression in the mutant bacteria

The mutant stock was streaked on TSB plates with the addition of 100 ppm kanamycin and cultured for over 18 h at 28°C. Subsequently, a single colony was cultured in 10 mL of TSB with 100 ppm kanamycin at 180 rpm and 28°C for 18–20 h. The cultured broth was added to 1 L of TSB and cultured until the OD at 600 nm reached 0.6. At this point (−10 h after the initial culture), l-arabinose (40% stock) was added to a final concentration of 0.2% to induce overexpression. The culture was further incubated at 180 rpm and 28°C for 72 h, including preculturing time, before the arabinose addition. For the uninduced bacterial culture, the same media and conditions were used for culturing the mutant bacteria, except for the addition of l-arabinose.

### 2.12 Nodulation assay

Hemocytic nodules, which are formed in response to the bacterial infection, were assessed using 3 days-old L5 larvae of *S. exigua*. The larva was injected with 1 μL of overnight-grown *E. coli* (5 × 10^7^ cells/mL) and 1 μL of bacterial extracts (100 ppm). A microsyringe (Hamilton, Reno, NV, United States) was used for the infection by thrusting the needle into the larval hemocoel through the proleg. The injected larvae were then incubated at 25°C for 8 h. After incubation, the larvae were dissected to count the melanized nodules under a microscope (Stemi SV11, Zeiss, Jena, Germany) at 50× magnification. Each treatment was replicated three times and consisted of five larvae per replication.

### 2.13 Hemocyte-spreading behavior assay

Hemolymph (approximately 250 μL) was collected from L5 larvae of *S. exigua* by cutting the proleg and mixing it with 350 μL of ice-cold ACB. The mixture was then subjected to centrifugation at 1,000× *g* for 2 min, and 400 μL of the supernatant was removed. The remaining hemocyte pellet was resuspended in TC-100 insect culture medium (HyClone, Daegu, Republic of Korea). A total of 10 μL of the reaction mixture, consisting of 9 μL of the hemocyte suspension and 1 μL of the bacterial extract (100 ppm), was mounted onto a glass slide. After incubation at room temperature under darkness for 30 min, hemocytes were observed under a phase contrast microscope (DM2500, Leica, Wetzlar, Germany) at 400× magnification. The spread hemocytes were characterized by cytoplasmic extensions beyond the cell boundary. Each treatment was replicated three times using independent hemocyte preparations. In each replication, 100 hemocytes were randomly selected for counting the spread hemocytes.

### 2.14 Measurement of phenoloxidase activity

Hemolymph was collected from L5 larvae of *S. exigua* and separated into hemocytes and plasma, as described above. Phenoloxidase (PO) activity in the plasma was measured using L-3,4-dihydroxyphenylalanine (DOPA) as a substrate. The 200 μL reaction mixture consisted of 10 μL of plasma, 10 μL of DOPA, 2 μL of bacterial metabolites (100 ppm), and 178 μL of PBS. The absorbance (ABS) of the reaction mixture was assessed at 495 nm using a VICTOR multi-label plate reader (PerkinElmer, Waltham, MA, United States). PO activity was quantified as ABS/min/mL. Each treatment was replicated three times.

### 2.15 Cytotoxicity analysis of the bacterial metabolites against Sf9 cells

An MTT assay was conducted using Sf9 cells according to the previously described protocol (Boonsuepsakul et al., 2008). Sf9 cells were seeded into 96-well plates at a density of 1.2 × 10^4^ cells per well and incubated for 24 h at 28°C. The cells were then treated with different bacterial metabolites and further incubated for 24 h at 28°C. After adding 10 μL of MTT solution (5 mg/mL in PBS) to each well, the cells were cultured for an additional 8 h at 28°C. Viable cells produced purple formazan granules dissolved in 50 μL of DMSO. The ABS of the resulting solution was measured at 570 nm using a microplate reader (Victor Multi-label Plate Reader, PerkinElmer).

### 2.16 Terminal deoxynucleotidyl transferase dUTP nick end labeling assay

The *in-situ* Cell Death Detection Kit from Abcam (Cambridge, United Kingdom) was used to perform the dUTP nick end labeling (TUNEL) assay on hemocytes of *S. exigua* L5 larvae. Larvae were injected with 2 μL of bacterial metabolite (100 ppm) and incubated for 18 h at 25°C. To prepare the hemocyte suspension, hemolymph was obtained from five or six L5 larvae and diluted in 300 μL of ACB. The sample was incubated on ice for 30 min before being replaced with TC-100 insect cell culture medium. For the assay, a reaction mixture was prepared by combining 10 μL of hemocyte suspension with 1 μL of a 10 μM solution of 5-bromo-2′-deoxyuridine (BrdU) containing terminal deoxynucleotidyl transferase (TdT). After the reaction mixture was prepared, it was placed on a cover glass in a wet chamber. To fix the cells, 2% paraformaldehyde was added, and the sample was incubated for 15 min. The cells were then washed with PBS and permeabilized with 0.3% Triton-X in PBS for 2 min at room temperature. To block non-specific binding sites, the cells were incubated with 4% bovine serum albumin in PBS for 10 min. Mouse anti-BrdU antibody (diluted 1:15 in blocking solution) was added, and the cells were incubated for 1 h at room temperature. After washing out the unbound anti-BrdU antibody, the FITC-conjugated anti-mouse IgG antibody (diluted 1:300 in blocking solution) was added, and the cells were incubated for 1 h at room temperature. DAPI (diluted 1:1,000 in PBS) was added to visualize the nuclei, and the cells were incubated at room temperature for 5 min. The cells were then washed with PBS, and a mixture of glycerol and PBS (1:1) solution was added to the cells on the cover glass. The cover glass was placed onto a glass slide, and the cells were observed under a fluorescence microscope (DM2500, Leica, Wetzlar, Germany) in FITC mode.

### 2.17 Bacterial virulence test

For this bioassay, L4 larvae of *S. exigua* were injected with freshly cultured bacteria at 1.5 × 10^2^ colony-forming unit (CFU)/larva using a microsyringe, as described above. Mortality was then measured at 72 h after the bacteria injection. Each treatment used 10 larvae and was replicated three times.

### 2.18 Data analysis

Virulence data were analyzed by arsine transformation (ANOVA). All assay data were analyzed using PROC GLM of the SAS program (SAS Institute Inc., 1989). All data were plotted as the mean ± standard error using Sigma Plot (Systat Software, Point Richmond, CA, United States). The least squared difference (LSD) test was used to compare the means with a type I error of 0.05.

## 3 Results

### 3.1 The *Photorhabdus temperata temperata* genome encodes 10 NRPS genes including *gxpS*

**Figure 1:**
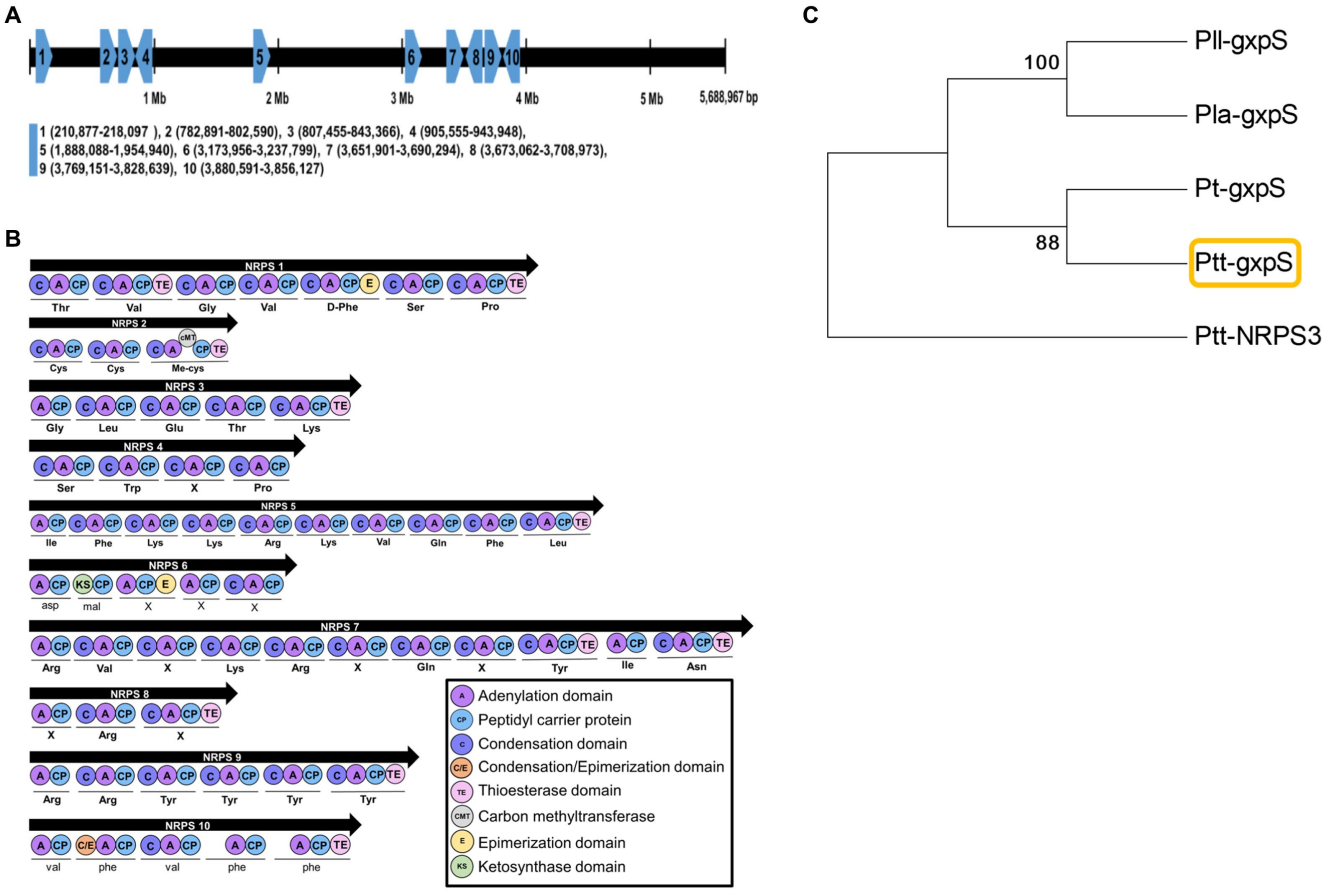
Prediction of GXP synthetase (*gxpS*) from the genome of *P. temperata temperata* (Ptt). **(A)** Relative localities of 10 non-ribosomal peptide synthetase (*NRPS 1* to *NRPS 10*) genes of Ptt on the genome of *P. luminescens laumondii* TT01 (GenBank accession number: NC_005126) by orthologous analysis (Supplementary Table S2). **(B)** Synthetic modules and their domain analyses of 10 *NRPS* genes using the antiSMASH bacterial version software (https://antismash.secondarymetabolites.org/). **(C)** A phylogenetic tree of *gxpS* genes in different *Photorhabdus* bacteria using the MEGA6 program. Bootstrap values were obtained with 1,000 repetitions to support branching and clustering. *P. luminescens laumondii* (Pll), *P. luminescens akhurstii* (Pla), *P. thracensis* (Pt), and Ptt had GenBank accession numbers of AXG48275.1, QXF34648.1, AKH65701.1, and JGVH01000001.1, respectively. Ptt-NRPS3 was retrieved with the accession number JGVH01000073.1.

A full genome (GenBank accession number: PRJNA217865) of *P. temperata temperata* Meg1 encodes 4,132 genes, in which 10 *NRPS* genes were predicted (Figure 1A). They were not clustered but located in different locations on the genome. Each *NRPS* gene had commonly adenylation, condensation, peptide carrier protein, and thioesterase domains (Figure 1B). However, their product peptides were predicted to be different. Among these NRPS genes, *NRPS10* was predicted to produce a pentapeptide (=GXP) and was called GXP synthetase (*gxpS*) in this bacterium. This *gxpS* shared amino acid sequences with those of other *gxpS* orthologs and was distinct from other *NRPS* genes (Figure 1C). Furthermore, the *gxpS* of the bacteria showed the highest sequence homology with *NRPS10* of *P. luminescens laumondii* TT01 (Supplementary Table S3), which was originally identified as *gxpS* (Nollmann et al., 2015).

### 3.2 *Photorhabdus temperata temperata* produce at least four different GXPs

**Figure 2:**
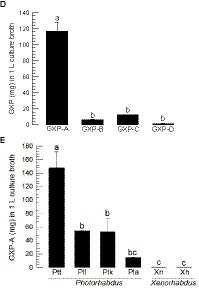
GXP synthetase (*gxpS*) expression and GXP production from *P. temperata temperata*. **(A)** Bacterial growth (measured by OD600) and *gxpS* expression in TSB media. The relative expression levels of *gxpS* were estimated by the fold changes compared to the expression level at 0 h. **(B)** Bacterial growth (measured by CFU) and *gxpS* expression in the hemocoel of *S. exigua*. **(C)** Chromatograms of four GXPs (GXP-A to GXP-D) from LC-MS/MS with their chemical structures and MS/MS spectrums. **(D)** Quantification of the four GXPs in the Ptt culture broth in TSB for 72 h. **(E)** Relative amounts of GXP-A in the culture broth produced by different entomopathogenic bacteria: *P. luminescens laumondii* (Pll), *P. luminescens kayaii* (Plk), *P. luminescens akhurstii* (Pla), *Xenorhabdus nematophila* (Xn), and *X. hominickii* (Xh). Each treatment was independently replicated three times. Different letters indicate significant differences among means at type I error = 0.05 (LSD test).

The *gxpS* expression was monitored at different culture periods during bacterial growth in TSB (Figure 2A) and *S. exigua* (Figure 2B). When grown in TSB, the bacteria exhibited exponential growth after an initial 6 h lag phase and reached stationary phase after 24 h. The *gxpS* exhibited a basal expression level during the exponential bacterial growth phase but rapidly increased its expression during the stationary phase. In *S. exigua*, the bacterial growth profile was similar to that in TSB, except a faster bacterial growth rate. Interestingly, *gxpS* was expressed at the early exponential stage in *S. exigua*.

GXP production was assessed from the culture broth of *P. temperata temperata* using four different GXP standards (GXP-A to GXP-D). These four standards were well separated in the pentafluorophenyl (F5) stationary phase, and their chemical identities were confirmed from MS/MS analyses (Figure 2C). Under the same LC-MS/MS analytical conditions, the four GXPs were detected in the culture broth extract of *P. temperata temperata* (Figure 2D). GXP-A was the most abundant among the four GXPs in the bacterial extract, with a concentration of approximately 135 mg/L. This value was over 7-fold higher than those of the other three GXP components.

GXP-A production was assessed in different *Photorhabdus* and *Xenorhabdus* bacteria (Figure 2E). Although it was detected in all test bacteria, it was produced significantly (*F* = 19.80; df = 5, 12; *p* < 0.0001) higher amounts in *Photorhabdus* than those in *Xenorhabdus*. Among the *Photorhabdus*, *P. temperata temperata* produced 2- to 3-fold more GXP-A than that of the other *Photorhabdus* species.

### 3.3 Generation of a mutant *Photorhabdus temperata temperata* exhibiting *gxpS* expression under an arabinose promoter

**Figure 3:**
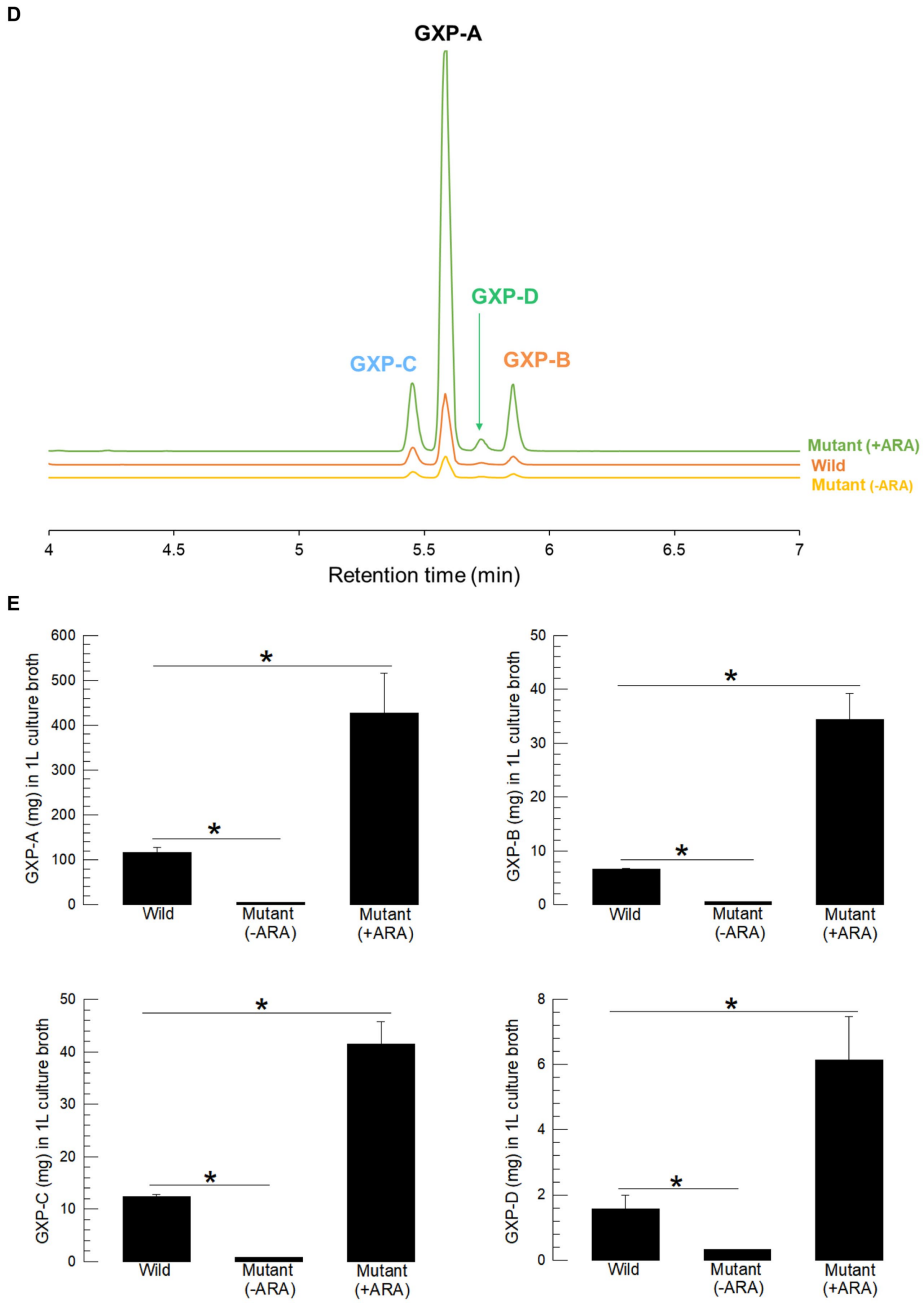
Promoter exchange of the *gxpS* gene of *P. temperata temperata* (Ptt) with an inducible promoter, araBAD, and manipulation of GXP synthesis. **(A)** Diagram illustrating the mutagenesis. The initial open reading frame region (600 bp) of *gxpS* was ligated into the pCEP vector at the araBAD promoter. The recombinant pCEP vector was inserted into *E. coli*-S17 competent cells. Subsequent bacterial conjugation between Ptt and *E. coli* led to homologous recombination at the overlapped sequence and the exchange of promoters. Arrows in the Ptt-mutant indicate the sequencing primers. **(B)** PCR products of the mutant with the sequencing primers. Wild type produced only a 500-bp PCR product, while the mutant gave two PCR products (500 and 5,823 bp). Sequencing results for the products are shown in Supplementary Figure S2. **(C)** Relative expression levels for the *gxpS* gene in wild-type and mutant bacteria. To induce *gxpS* expression in the mutant bacteria, 0.2% of l-arabinose (ARA) was added to the culture broth when the OD600 of the bacterial culture reached −0.6. The symbols “–” and “+” indicate the absence or addition of ARA, respectively. **(D)** A representative chromatogram of LC-MS/MS showing four GXPs in the three different bacterial culture broths. **(E)** Comparison of four GXP amounts in the three different bacterial culture broths. The bacteria were cultured in TSB at 28°C for 72 h. Each treatment was independently replicated three times. Asterisks indicate significant differences between two means at type I error = 0.05 (LSD test).

To confirm the role of *gxpS* expression in the production of GXP in *P. temperata temperata*, its mutant was constructed by replacing the original bacterial promoter with an inducible arabinose promoter (Figure 3A). A partial (600 bp) open reading frame at the 5′ end was cloned into a pCEP vector with an arabinose promoter. The recombinant vector was transferred to the wild type of *P. temperata temperata* through conjugation and inserted into the bacterial genome by homologous recombination. The mutant was confirmed by long-range PCR, which produced 5,823 bp covering the insertion sites (Figure 3B). The PCR product was sequenced and showed the insertion sites in addition to the vector (Supplementary Figure S2). The mutant bacteria showed altered expression levels of *gxpS* according to the presence of the inducer, l-arabinose (Figure 3C). Without the inducer, the mutant showed a significantly (*F* = 15.55; df = 1, 4; *p* = 0.0169) lower level of *gxpS* expression compared to that of the wild type. In contrast, the addition of the inducer significantly (*F* = 24.49; df = 1, 4; *p* = 0.0078) upregulated *gxpS* expression compared to the wild type.

Inducible *gxpS* expression led to the modulation of GXP production in the mutant bacteria (Figure 3D). Without the inducer, the mutant produced a significantly (*F* = 97.49; df = 1, 4; *p* = 0.0006) lower GXP-A amount compared to that of the wild type. In contrast, the addition of the inducer significantly (*F* = 12.13; df = 1, 4; *p* = 0.0253) increased GXP-A production compared to the wild type (Figure 3E). Similar modulations were detected in the other three GXPs in the mutant bacteria.

### 3.4 Control of *gxpS* expression led to an alteration in the immunosuppressive activity of *Photorhabdus temperata temperata* against the target insect

**Figure 4:**
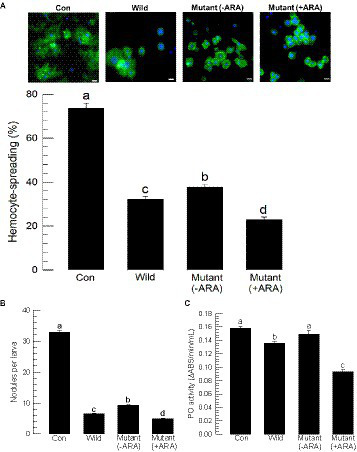
Alteration of the immunosuppressive activities of *P. temperata temperata* (Ptt) by manipulating the expression level of the *gxpS* gene in *S. exigua*. “Wild” represents the *gxpS* promoter of Ptt without any exchange. “Mutant” represents the exchanged *gxpS* promoter with the araBAD promoter. To induce *gxpS* expression in the mutant bacteria, 0.2% of l-arabinose (ARA) was added to the culture broth when the OD600 of the bacterial culture reached −0.6. The symbols “–” or “+” indicate the absence or addition of ARA, respectively. All assessments used the organic extracts of the bacterial culture broth. **(A)** Hemocyte-spreading assay observed by F-actin growth in response to the bacterial extract. Hemocytes were examined under a fluorescence microscope at 200× magnification. F-actin filaments were specifically recognized by FITC-tagged phalloidin (green), and the nucleus was stained with DAPI (blue). “Con” indicates solvent (DMSO) treatment. Scale bar represents 10 μm. **(B)** Nodulation assay. The number of nodules was counted after 8 h post-injection. **(C)** Phenoloxidase (PO) activity analysis. Each treatment was independently replicated three times. Different letters indicate significant differences among means at type I error = 0.05 (LSD test).

The metabolite extracts of the wild-type and mutant bacteria contained different amounts of GXPs. All bacterial extracts significantly suppressed the cellular immune response, as measured by hemocyte-spreading behavior (Figure 4A). However, there was variation among the bacterial extracts, in which the mutant extract (−ARA) without the inducer was less potent in inhibiting the immune responses compared to the wild-type extract, while the mutant extract (+ARA) with the addition of the inducer became more potent. Similar phenomena were observed in the assessments of other immune responses as measured by nodule formation (Figure 4B) and PO activity (Figure 4C).

### 3.5 Control of *gxpS* expression led to an alteration in the cytotoxicity of *Photorhabdus temperata temperata* against target insect hemocytes

**Figure 5:**
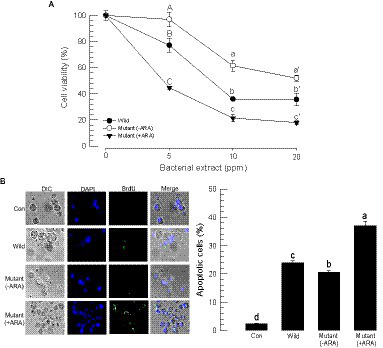
Alteration of the cytotoxic activities of *P. temperata temperata* (Ptt) by manipulating the expression level of the *gxpS* gene in *S. exigua*. “Wild” represents the *gxpS* promoter of Ptt without any exchange. “Mutant” represents the exchanged *gxpS* promoter with the araBAD promoter. To induce *gxpS* expression in the mutant bacteria, 0.2% of l-arabinose (ARA) was added to the culture broth when the OD600 of the bacterial culture reached −0.6. The symbols “–” and “+” indicate the absence or addition of ARA, respectively. All assessments used the organic extracts of the bacterial culture broth. **(A)** MTT assay of the bacterial extracts against Sf9 cells. The cells were exposed to bacterial extract at different doses for 24 h at 28°C. After 4 h of incubation with MTT, the resulting formazan granules were measured at 570 nm. Cell viability was measured by relative absorbance compared to the cells at 0 ppm. Each treatment was replicated three times. Different letters above the standard deviation bars indicate significant (*p* < 0.05, type I error) differences among means at each bacterial dose. **(B)** Apoptosis analysis of the hemocytes of *S. exigua* in the bacterial extracts using a TUNEL assay. At 18 h after the injection of the bacterial extracts (100 ppm), hemocytes were collected and labeled with a 5-bromouridine (BrdU) solution containing TdT. The specific antibody against BrdU was added for binding to the labeled DNA, and then the antibody complex was detected with a secondary antibody conjugated with FITC. The nucleus was stained with DAPI. Apoptotic cells responding to the BrdU antibody were counted among 100 randomly chosen cells. Each treatment was independently replicated three times. Different letters indicate significant differences among means at type I error = 0.05 (LSD test).

Both wild-type and mutant bacterial extracts exhibited cytotoxicity against insect cells (Figure 5). Using an MTT test, the bacterial extracts were applied to Sf9 cells, which resulted in significant cytotoxicity in a dose-dependent manner (Figure 5A). However, the cytotoxicity was varied among the bacterial extract, in which the mutant extract (−ARA) without the inducer exhibited less potent cytotoxicity compared to the wild-type extract, whereas the mutant extract (+ARA) with the addition of the inducer became more potent.

Cytotoxic activity was also detected in hemocytes through apoptosis. A TUNEL assay showed that the bacterial extracts induced DNA fragmentation (see BrdU-positive cells) in the hemocytes (Figure 5B). However, the apoptotic activity was varied among the bacterial extract, in which the mutant extract (−ARA) without the inducer exhibited less potent apoptotic activity compared to the wild-type extract, whereas the mutant extract (+ARA) with the addition of the inducer became more potent.

### 3.6 Control of *gxpS* expression led to the insecticidal activity of *Photorhabdus temperata temperata*

**Figure 6:**
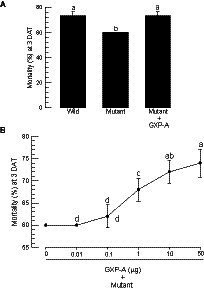
Comparison of wild-type and mutant *P. temperata temperata* (Ptt) for insecticidal activities against *S. exigua*. “Wild” represents the *gxpS* promoter of Ptt without any exchange. “Mutant” represents the exchanged *gxpS* promoter with the araBAD promoter. L4 larvae were injected with 1 μL volume containing 1.5 × 10^2^ CFU per larva and incubated for 3 days after treatment (DAT). Each treatment used 10 larvae and was replicated three times. **(A)** Insecticidal activities of wild-type and mutant bacteria. GXP-A (10 μg/larva) was added along with the bacteria. **(B)** Dose mortality curve of GXP-A addition to the mutant bacteria (1.5 × 10^2^ CFU/larva). Different letters above the standard deviation bars indicate significant differences among means at type I error = 0.05 (LSD test).

Bacterial virulence was assessed between the wild-type and the mutant bacteria against *S. exigua* by injecting the bacteria into the larval hemocoel (Figure 6). Arabonose is rarely detected in insects. Exogenous arabinose to the insect was not likely to be used by the injected bacteria. Thus, the experiment should assess the *gxpS* deletion mutant because no arabinose did not induce this gene expression. To rescue the mutant, this study used the exogenous addition of GXP-A. All bacteria, including the wild type, resulted in significant insecticidal activity within 3 days. However, the mutant showed significantly lower insecticidal activities compared to those of the wild type (Figure 6A). When the mutant was injected along with GXP-A at 10 μg/larva, the insecticidal activity was significantly increased in a dose-dependent manner (Figure 6B). GXP-A was produced in TSB at 140 mg/L. *S. exigua* larvae at fifth instar have −100 μL of hemolymph. Thus, 14 μg of GXP-A may be produced in the larvae infected with these bacteria. Thus, we used the test dose of 10 μg/larva.

## 4 Discussion

Although a number of bacterial secondary metabolites are species- or genus specifically produced, GXPs are known to be commonly produced by *Xenorhabdus* and *Photorhabdus* (Tobias et al., 2017). For example, a wide collection of these bacteria from their host nematodes in a reserved national park in a subtropical region showed that all isolates identified as *Xenorhabdus* and *Photorhabdus* had GXP derivatives (Muangpat et al., 2017). Thus, we hypothesized that *P. temperata temperata* isolate produces GXP derivatives and investigated its genetic component. Our results bolstered the hypothesis through bioinformatics and chemical analysis. First, a specific *gxpS* gene was predicted from NRPS genes encoded in *P. temperata temperata* genome. Second, LC-MS/MS analysis identified the four different GXP compounds from the bacterial culture broth. Third, the mutagenesis of *gxpS* promoter allowed us to manipulate the GXP production of the bacteria. Fourth, the manipulation of *gxpS* expression led to alteration of host insect immunity.

The GXP synthetase gene, *gxpS*, was predicted from the bacterial genome of *P. temperata temperata* and expressed in the target insect, *S. exigua*, at the early infection stage. The modules of the gene were predicted to synthesize pentapeptide(s) without the catalytic activity of the ribosome and shared high homologies with the orthologs of other *Photorhabdus* species. Its expression profile appeared to follow the bacterial growth phases of *P. temperata temperata*, in which it was expressed in the stationary phase during culture in TSB. However, its expression was different in the target insect, *S. exigua*, in which it was expressed at the early infection stage. This suggests that an insect host signal may trigger *gxpS* expression. *Photorhabdus* and *Xenorhabdus* exhibit a similar life cycle, with a mutual form in the nematode host and a pathogenic form in the insect host, through a convergent evolution (Chaston et al., 2011). These two life forms possess different bacterial morphological and behavioral characteristics in Photorhabdus and are interconvertible by the stochastic inversion of the promoter to control the gene expression of maternal adhesion (*Mad*) fimbriae (Somvanshi et al., 2012). The conversion from mutualistic to pathogenic form leads to expression of the various genes associated with bacterial virulence against the insect hosts.

To examine the gene function, *gxpS* expression was manipulated by promoter exchange with an inducible arabinose promoter, according to the method of Bode et al. (2015). The mutant bacteria expressed *gxpS* under the control of the l-arabinose inducer, in which *gxpS* was expressed at a low level without the inducer but a high level of gene expression was detected after inducer addition. This suggested that the *gxpS* expression of the mutant was controlled by the inducible arabinose promoter. Interestingly, all four GXPs were produced in small quantities in the culture broth of the mutant bacteria without l-arabinose, while they were produced in large amounts in the culture broth of the mutant bacteria after the addition of the inducer. These strongly support that the predicted *gxpS* is responsible for GXP production in *P. temperata temperata*.

Four GXPs (GXP-A to GXP-D) were identified from the culture broth of *P. temperata temperata*. GXP-A was the dominant type, while the other three GXPs were produced at much lower levels. GXP-A is an immunosuppressant since it suppresses the cellular immune responses of *S. exigua* (Shi et al., 2022). Furthermore, *P. temperata temperata* may produce other types of GXPs (GXP-E to GXP-H), which contain different building blocks such as p-aminophenylalanine (PAPA) and its monomethyl derivative (MMPAPA), as demonstrated in *Photorhabdus luminescens* TTO1 (Nollmann et al., 2015). The PAPA operon containing *plu-35667-plu3561*, which is conserved in *Photorhabdus* but not in *Xenorhabdus*, controls the biosynthesis of PAPA and MMPAPA. This operon activates the gene expression within insect hosts, although the host factor remains unknown (Nollmann et al., 2015). This suggests that other types of GXPs may be produced in *P. temperata temperata*.

The organic extracts formed in the bacterial culture broth used for GXP quantification showed difference in the immunosuppression of *S. exigua* between the wild-type and mutant bacteria. The extract from the wild-type bacteria suppressed hemocyte-spreading behavior and nodule formation in response to bacterial infection. PO activity was especially suppressed by the bacterial extract. In contrast, the bacterial extract from the mutant without the inducer showed much less potent activity in suppression of the cellular immune responses, whereas the bacterial extract of the mutant induced by l-arabinose highly suppressed the cellular immune responses. Nodule formation has been regarded as a cellular immune response in insects (Lavine and Strand, 2002). It requires hemocyte-spreading behavior to trap the infective bacteria or other pathogens by cytoplasmic extension (Ratcliffe and Gagen, 1977; Lapointe et al., 2012). Subsequent melanization mediated by PO activity forms the black nodules by the cross-linking of oxygenated catecholamine compounds associated with proteins (Cerenius et al., 2008). Hemocyte-spreading behavior is mediated by eicosanoids such as prostaglandins (PGs) via cytoskeletal rearrangement to extend the cytoplasm and aquaporin activation to increase the local volume of the hemocytes (Ahmed and Kim, 2021). Furthermore, PO activation is mediated by PGs via stimulating the release of inactive PO from oenocytoids to the plasma, in which PO is activated by proteolytic cleavage (Shrestha and Kim, 2008). These findings support the immunosuppressive role of GXP in insects (Shi et al., 2022). The early induction of *gxpS* expression in *S. exigua* infected with *P. temperata temperata* suggests its role in the early infection stage by establishing the host immunosuppressive conditions for bacterial survival and growth against the insect immune responses. Indeed, eicosanoids mediate the early immune responses in *S. exigua*, but their biosynthesis is acutely suppressed by bacterial infection with *P. temperata temperata* through inhibiting phospholipase A_2_ (PLA_2_), which catalyzes the committed step for eicosanoid biosynthesis (Kim et al., 2018). This suggests that the early induction of *gxpS* expression inhibits the insect PLA_2_, which results in a fatal immunosuppressive state in the insect host. This condition would be favored by the bacterium and its symbiotic host nematode, *Heterorhabditis megidis*, for successful parasitism.

The mutant analysis of *P. temperata temperata* by modulating *gxpS* promoter activity indicated the role of GXP in inducing hemolytic activity by inducing apoptosis. Compared to the wild-type bacterial extract, the mutant bacterial extracts altered the hemolytic activity depending on the GXP concentrations. The molecular mechanism that induces apoptosis and the subsequent cytotoxic activity remains unknown.

Immunosuppression was crucial to express the bacterial virulence of *P. temperata temperata* against the target insect, *S. exigua*. Compared to the wild-type bacteria, the mutants exhibited a significant loss of virulence when they infected the host larvae. In contrast, the addition of GXP rescued the lost virulence. These findings suggest that immunosuppression plays a crucial role in bacterial virulence. The relationship between immunosuppressive activity and bacterial virulence was demonstrated in a related bacterium, *Xenorhabdus nematophila* (Hasan et al., 2019). In this study, the immunosuppressive activities and virulence of six different *X. nematophila* strains were compared against those of *S. exigua*. All six strains suppressed PLA_2_ activity but showed differential inhibitory activities. The difference in the inhibitory activity of PLA_2_ was highly correlated with immunosuppressive activity and virulence. PLA_2_ is the common target of both *Xenorhabdus* and *Photorhabdus* for their pathogenesis (Kim et al., 2005). Furthermore, Ahmed and Kim (2018) showed that the differential inhibitory activity of PLA_2_ is correlated with bacterial virulence. These findings suggest that the immunosuppression induced by *P. temperata temperata* is functionally related to the bacterial virulence. Shi et al. (2022) demonstrated that GXP-A suppresses the nodule formation of *S. exigua*. The immunosuppressive activity of GXP would contribute to bacterial virulence in *P. temperata temperata*.

Altogether, our results support the GXP production from *P. temperata temperata*. Thus, the study also suggests that GXPs are produced by the catalytic activity of the *gxpS* gene product. Based on the physiological function of GXP-A, our study suggests that other GXPs may cooperatively mediate the host manipulation. This should be explored in a subsequent study.

## Data availability statement

The datasets presented in this study can be found in online repositories. The names of the repository/repositories and accession number(s) can be found in the article/Supplementary material.

## Ethics statement

The requirement of ethical approval was waived by Animal Ethics Committee of Andong National University for the studies involving animals because small insects are used for this experiment. The studies were conducted in accordance with the local legislation and institutional requirements.

## Author contributions

GJ: Data curation, Formal analysis, Methodology, Software, Validation, Visualization, Writing – original draft. MH: Data curation, Formal analysis, Methodology, Software, Validation, Visualization, Writing – original draft. DH-L: Data curation, Formal analysis, Methodology, Validation, Resources, Visualization. I-HK: Data curation, Formal analysis, Investigation, Methodology, Software, Validation, Visualization, Writing – original draft. J-SJ: Data curation, Formal analysis, Investigation, Methodology, Software, Validation, Visualization, Writing – original draft. HB: Conceptualization, Resources, Supervision, Validation, Writing – review & editing. YK: Conceptualization, Funding acquisition, Investigation, Project administration, Resources, Supervision, Writing – original draft, Writing – review & editing.

## Footnotes

^1^ https://antismash.secondarymetabolites.org/

^2^ https://www.megasoftware.net/

^3^ https://blast.ncbi.nlm.nih.gov/

## References

[ref1] Ahmed S. Kim Y. (2018). Differential immunosuppression by inhibiting PLA_2_ affects virulence of *Xenorhabdus hominickii* and *Photorhabdus temperata temperata*. J. Invertebr. Pathol. 157, 136–146. doi: 10.1016/j.jip.2018.05.009, PMID: 29802883

[ref2] Ahmed S. Kim Y. (2021). PGE_2_ mediates hemocyte-spreading behavior by activating aquaporin via cAMP and rearranging actin cytoskeleton via Ca^2+^. Dev. Comp. Immunol. 125:104230. doi: 10.1016/j.dci.2021.104230 34388674

[ref3] Bode E. Brachmann A. O. Kegler C. Simsek R. Dauth C. Zhou Q.. (2015). Simple “on-demand” production of bioactive natural products. Chembiochem 16, 1115–1119. doi: 10.1002/cbic.201500094, PMID: 25826784

[ref4] Bode H. B. Reimer D. Fuchs S. W. Kirchner F. Dauth C. Kegler C.. (2012). Determination of the absolute configuration of peptide natural products by using stable isotope labeling and mass spectrometry. Chemistry 18, 2342–2348. doi: 10.1002/chem.201103479 22266804

[ref5] Boemare N. E. (2002). “Biology taxonomy and systematics of *Photorhabdus* and *Xenorhabdus*” in Entomopathogenic nematology. ed. Gaugler R. (Wallingford, UK: CABI), 35–56.

[ref6] Boonsuepsakul S. Luepromchai E. Rongnoparut P. (2008). Characterization of *Anopheles minimus* CYP6AA3 expressed in a recombinant baculovirus system. Arch. Insect Biochem. Physiol. 69, 13–21. doi: 10.1002/arch.20248 18615616

[ref7] Bustin S. A. Benes V. Garson J. A. Hellemans J. Huggett J. Kubista M.. (2009). The MIQE guidelines: minimum information for publication of quantitative real-time PCR experiments. Clin. Chem. 55, 611–622. doi: 10.1373/clinchem.2008.112797 19246619

[ref8] Cerenius L. Lee B. L. Söderhäll K. (2008). The proPO-system: pros and cons for its role in invertebrate immunity. Trends Immunol. 29, 263–271. doi: 10.1016/j.it.2008.02.009, PMID: 18457993

[ref9] Chaston J. M. Suen G. Tucker S. L. Andersen A. W. Bhasin A. Bode E.. (2011). The entomopathogenic bacterial endosymbionts *Xenorhabdus* and *Photorhabdus*: convergent lifestyles from divergent genomes. PLoS One 6:e27909. doi: 10.1371/journal.pone.0027909, PMID: 22125637 PMC3220699

[ref10] Cimen H. Touray M. Gulsen S. H. Hazir S. (2022). Natural products from *Photorhabdus* and *Xenorhabdus*: mechanisms and impacts. Appl. Microbiol. Biotechnol. 106, 4387–4399. doi: 10.1007/s00253-022-12023-9, PMID: 35723692

[ref11] Goh H. G. Park J. D. Choi Y. M. Choi K. M. Park I. S. (1991). The host plants of beet armyworm, *Spodoptera exigua* (Hübner), (Lepidoptera: Noctuidae) and its occurrence. Korean J. Appl. Entomol. 30, 111–116.

[ref12] Hasan M. A. Ahmed S. Mollah M. M. I. Lee D. Kim Y. (2019). Variation in pathogenicity of different strains of *Xenorhabdus nematophila*; differential immunosuppressive activities and secondary metabolite production. J. Invertebr. Pathol. 166:107221. doi: 10.1016/j.jip.2019.107221, PMID: 31356819

[ref13] Hrithik M. T. H. Park Y. Park H. Kim Y. (2022). Integrated biological control using a mixture of two entomopathogenic bacteria, *Bacillus thuringiensis* and *Xenorhabdus hominickii*, against *Spodoptera exigua* and other congeners. Insects 13:860. doi: 10.3390/insects13100860, PMID: 36292808 PMC9604179

[ref14] Kang S. Han S. Kim Y. (2004). Identification of an entomopathogenic bacterium, *Photorhabdus temperata* subsp. *temperata*, in Korea. J. Asia Pac. Entomol. 7, 331–337. doi: 10.1016/S1226-8615(08)60235-6

[ref15] Kim Y. Ahmed S. Stanley D. An C. (2018). Eicosanoid-mediated immunity in insects. Dev. Comp. Immunol. 83, 130–143. doi: 10.1016/j.dci.2017.12.005, PMID: 29225005

[ref16] Kim Y. Ji D. Cho S. Park Y. (2005). Two groups of entomopathogenic bacteria, *Photorhabdus* and *Xenorhabdus*, share an inhibitory action against phospholipase A_2_ to induce host immunodepression. J. Invertebr. Pathol. 89, 258–264. doi: 10.1016/j.jip.2005.05.001, PMID: 15979640

[ref17] Lapointe J. F. Dunphy G. B. Mandato C. A. (2012). Hemocyte-hemocyte adhesion and nodulation reactions of the greater wax moth, *Galleria mellonella* are influenced by cholera toxin and its B-subunit. Results Immunol. 2, 54–65. doi: 10.1016/j.rinim.2012.02.002, PMID: 24371567 PMC3862387

[ref18] Lavine M. D. Strand M. R. (2002). Insect hemocytes and their role in immunity. Insect Biochem. Mol. Biol. 32, 1295–1309. doi: 10.1016/S0965-1748(02)00092-9 12225920

[ref19] Livak K. J. Schmittgen T. D. (2001). Analysis of relative gene expression data analysis using real-time quantitative PCR and the 2^−ΔΔCT^ method. Methods 25, 402–408. doi: 10.1006/meth.2001.1262 11846609

[ref20] Muangpat P. Yooyangket T. Fukruksa C. Suwannaroj M. Yimthin T. Sitthisak S.. (2017). Screening of the antimicrobial activity against drug resistant bacteria of *Photorhabdus* and *Xenorhabdus* associated with entomopathogenic nematodes from Mae Wong National Park, Thailand. Front. Microbiol. 8:1142. doi: 10.3389/fmicb.2017.01142, PMID: 28702004 PMC5487437

[ref21] Neubacher N. Tobias N. J. Huber M. Cai X. Glatter T. Pidot S. J.. (2020). Symbiosis, virulence and natural-product biosynthesis in entomopathogenic bacteria are regulated by a small RNA. Nat. Microbiol. 5, 1481–1489. doi: 10.1038/s41564-020-00797-5, PMID: 33139881 PMC7610847

[ref22] Nollmann F. I. Dauth C. Mulley G. Kegler C. Kaiser M. Waterfield N. R.. (2015). Insect-specific production of new GameXPeptides in *Photorhabdus luminescens* TTO1, widespread natural products in entomopathogenic bacteria. Chembiochem 16, 205–208. doi: 10.1002/cbic.201402603, PMID: 25425189

[ref23] Park Y. Kang S. Sadekuzzaman M. Kim H. Jung J. K. Kim Y. (2017). Identification and bacterial characteristics of *Xenorhabdus hominickii* ANU101 from an entomopathogenic nematode, *Steinernema monticolum*. J. Invertebr. Pathol. 144, 74–87. doi: 10.1016/j.jip.2017.02.002, PMID: 28193447

[ref24] Park Y. Kim Y. (2000). Eicosanoids rescue *Spodoptera exigua* infected with *Xenorhabdus nematophilus*, the symbiotic bacteria to the entomopathogenic nematode *Steinernema carpocapsae*. J. Insect Physiol. 46, 1469–1476. doi: 10.1016/S0022-1910(00)00071-8 10891575

[ref25] Ratcliffe N. A. Gagen S. J. (1977). Studies on the *in vivo* cellular reactions of insects: an ultrastructural analysis of nodule formation in *Galleria mellonella*. Tissue Cell 9, 73–85. doi: 10.1016/0040-8166(77)90050-7, PMID: 408940

[ref26] SAS Institute Inc.. (1989) SAS/STAT user’s guide, Release 6.03, Cary, NC; SAS Institute Inc.

[ref27] Seo S. Lee S. Hong Y. Kim Y. (2012). Phospholipase A_2_ inhibitors synthesized by two entomopathogenic bacteria, *Xenorhabdus nematophila* and *Photorhabdus temperata* subsp. *temperata*. Appl. Environ. Microbiol. 78, 3816–3823. doi: 10.1128/AEM.00301-12, PMID: 22447611 PMC3346408

[ref28] Shi Y. M. Bode H. B. (2018). Chemical language and warfare of bacterial natural products in bacteria-nematode-insect interactions. Nat. Prod. Rep. 35, 309–335. doi: 10.1039/C7NP00054E, PMID: 29359226

[ref29] Shi Y. M. Hirschmann M. Shi Y. N. Ahmed S. Abebew D. Tobias N. J.. (2022). Global analysis of biosynthetic gene clusters reveals conserved and unique natural products in entomopathogenic nematode-symbiotic bacteria. Nat. Chem. 14, 701–712. doi: 10.1038/s41557-022-00923-2, PMID: 35469007 PMC9177418

[ref30] Shrestha S. Kim Y. (2008). Eicosanoids mediate prophenoloxidase release from oenocytoids in the beet armyworm *Spodoptera exigua*. Insect Biochem. Mol. Biol. 38, 99–112. doi: 10.1016/j.ibmb.2007.09.013, PMID: 18070669

[ref31] Somvanshi V. S. Sloup R. E. Crawford J. M. Martin A. R. Heidt A. J. Kim K. S.. (2012). A single promoter inversion switches *Photorhabdus* between pathogenic and mutualistic states. Science 337, 88–93. doi: 10.1126/science.1216641, PMID: 22767929 PMC4006969

[ref32] Stock S. P. (2019). Partners in crime: symbiont-assisted resource acquisition in Steinernema entomopathogenic nematodes. Curr. Opin Insect. Sci. 32, 22–27. doi: 10.1016/j.cois.2018.10.006, PMID: 31113627

[ref33] Tobias N. J. Wolff H. Djahanschiri B. Grundmann F. Kronenwerth M. Shi Y. M.. (2017). Natural product diversity associated with the nematode symbionts *Photorhabdus* and *Xenorhabdus*. Nat. Microbiol. 2, 1676–1685. doi: 10.1038/s41564-017-0039-9 28993611

[ref34] Yimthin T. Fukruksa C. Muangpat P. Dumidae A. Wattanachaiyingcharoen W. Vitta A.. (2021). A study on *Xenorhabdus* and *Photorhabdus* isolates from northeastern Thailand: identification, antibacterial activity, and association with entomopathogenic nematode hosts. PLoS One 16:e0255943. doi: 10.1371/journal.pone.0255943 34383819 PMC8360611

