# Supplementary material for: Manipulation of GameXPeptide synthetase gene expression by a promoter exchange alters the virulence of an entomopathogenic bacterium, Photorhabdus temperata temperata, by modulating insect immune responses
Source: Front Microbiol. 2023 Dec 18;14:1271764. doi: 10.3389/fmicb.2023.1271764 (PMC10764021; doi:10.3389/fmicb.2023.1271764)
Supplement: Supplementary file 1 [file Data_Sheet_1.docx]

**Supplementary information**

**Table S1**. GenBank accession numbers 10 *NRPS* genes of *P. temperata temperata* (Ptt) and their locations on the genome with a reference genome of *P. luminescens laumondii* TT01 (Pll)

**Table S2**. List of primers used in this study

**Table S3.** Sequence homology search of *gxpS* (GenBank accession number: AXG48275.1) encoded in *P. luminescens laumondii* TT01 to 10 NRPS genes encoded in *P. temperata temperata* (Ptt) genome using NCBI-BLAST.

**Fig. S1.** Typical chromatograms and purity estimation of the synthesized GXPs (**A-D**) using LC-UV by manufacturer. Arrows indicate the purity of the synthesized peptides.

**Fig. S2.** Sequence analysis of the *gxpS* mutant of *P. temperata temperata* (Ptt). Gray color represents wild type of Ptt promoter region, blue color shows wild type Ptt *gxpS* region, purple color shows araBAD promoter, and yellow color shows pBAD *gxpS* region.

**Table S1**. GenBank accession numbers 10 *NRPS* genes of *P. temperata temperata* (Ptt) and their locations on the genome with a reference genome of *P. luminescens laumondii* TT01 (Pll)

| Gene | GenBank accession numbers | *Ptt* | *Pll* | NCBI BLAST | | | |
| --- | --- | --- | --- | --- | --- | --- | --- |
|  |  | Location in scaffold | Location in  genome | Max  score | E  value | | Homology (%) |
| *NRPS 1* | JGVH01000010.1 | 9499-16719 | 210,877-218,097 | 9122 | 0 | | 87.9 |
| *NRPS 2* | JGVH01000006.1 | 47685-67384 | 782,891-802,590 | 16059 | 0 | 86.9 | |
| *NRPS 3* | JGVH01000073.1 | 1-36,120 | 807,455-843,366 | 10910 | 0 | | 88.4 |
| *NRPS 4* | JGVH01000062.1 | 1-34725 | 905,555-943,948 | 3956 | 0 | | 85.5 |
| *NRPS 5* | JGVH01000021.1 | 1-66852 | 1,888,088-1,954,940 | 11464 | 0 | | 86.66 |
| *NRPS 6* | JGVH01000019.1 | 1836-65678 | 3,173,956-3,237,799 | 12339 | 0 | | 86.66 |
| *NRPS 7* | JGVH01000070.1 | 1-38,393 | 3,651,901-3,690,294 | 9282 | 0 | | 82.0 |
| *NRPS 8* | JGVH01000074.1 | 1-35,911 | 3,673,062-3,708,973 | 9638 | 0 | | 79.8 |
| *NRPS 9* | JGVH01000009.1 | 34551-94039 | 3,769,151-3,828,639 | 11183 | 0 | | 87.40 |
| *NRPS 10* | JGVH01000001.1 | 115708-131217 | 3,880,591-3,856,127 | 13605 | 0 | | 79.43 |

**Table S2**. List of primers used in this study

| Genes | Primers (5’→3’) | Annealing temperature (℃) | Product size (bp) |
| --- | --- | --- | --- |
| Ptt-GXP-Nde1-F | CATATGATGAAAGACAGTATTACCAG | 55 | 600 |
| Ptt-GXP-Pst1-R | CTGCAGCATGATATACGCCGGCCCGG |  |  |
| Ptt-Mutant-long-F | CGGATGTAACCGGGCTATT | 53 | 5,823 |
| Ptt-Mutant-long-R | GCCACTACCAGTTCAACCGA |  |  |
| Ptt-Mutant-confirm-F1 | TATGCGGATGTAACCGGGC | 55 | 1,196 |
| Ptt-Mutant-confirm-R1 | ATCTGAAGATCAGCAGTTCAACCT |  |  |
| Ptt-Mutant-confirm-F2 | ACCAATTGTCCATATTGCATCAGA | 55 | 803 |
| Ptt-Mutant-confirm-R2 | CGGCACTGGAACCCAATAAA |  |  |
| Ptt-gxpS-QPCR-F | AAATCGCACTCAGGTGCTCA | 55 | 277 |
| Ptt-gxpS-QPCR-R | GTATCTGGTGCGCGGATGTA |  |  |
| 16S-QPCR-F | ACTGAGACACGGCCCAGACTC | 55 | 250 |
| 16S-QPCR-R | CGCCCAGTCATTCCGATTAAC |  |  |

**Table S3.** Sequence homology search of *gxpS* (GenBank accession number: AXG48275.1) encoded in *P. luminescens laumondii* TT01 to 10 NRPS genes encoded in *P. temperata temperata* (Ptt) genome using NCBI-BLAST.

| Ptt | | NCBI BLAST | | | | |
| --- | --- | --- | --- | --- | --- | --- |
| Genes | Contig | Max  score | E-value | | | Homology (%) |
| *NRPS 1* | JGVH01000010.1 | - | - | | | - |
| *NRPS 2* | JGVH01000006.1 | - | - | - | - | |
| *NRPS 3* | JGVH01000073.1 | - | - | | | - |
| *NRPS 4* | JGVH01000062.1 | - | - | | | - |
| *NRPS 5* | JGVH01000021.1 | 3533 | 0.0 | | | 74.78 |
| *NRPS 6* | JGVH01000019.1 | - | - | | | - |
| *NRPS 7* | JGVH01000070.1 | 1953 | 0.0 | | | 76.61 |
| *NRPS 8* | JGVH01000074.1 | 263 | 3e-71 | | | 74.56 |
| *NRPS 9* | JGVH01000009.1 | 2324 | 0.0 | | | 75.41 |
| *NRPS 10* | JGVH01000001.1 | 8839 | 0.0 | | | 80.13 |

**A**

**
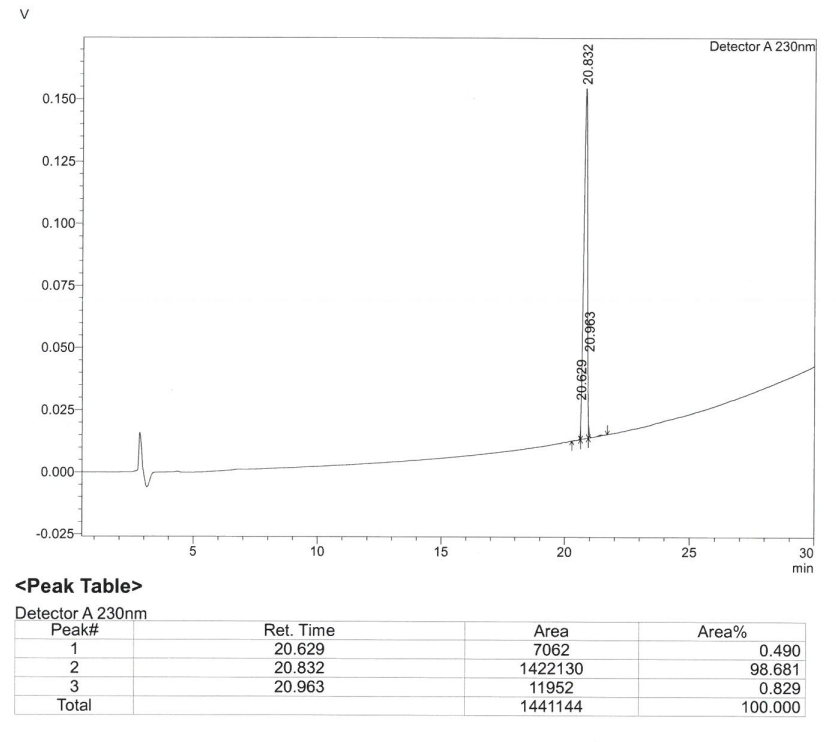
**

**B**

**
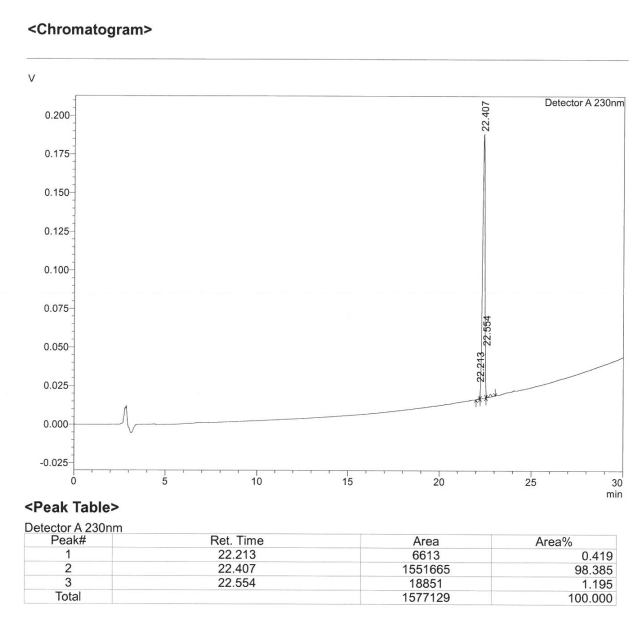
**

**C**

**
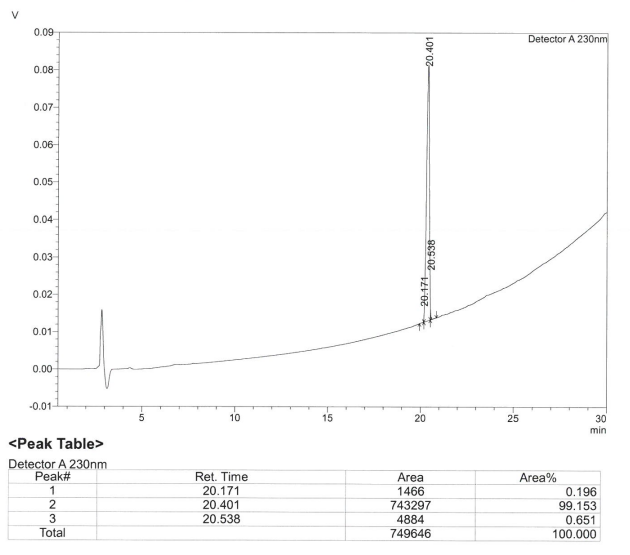
**

**D**

**
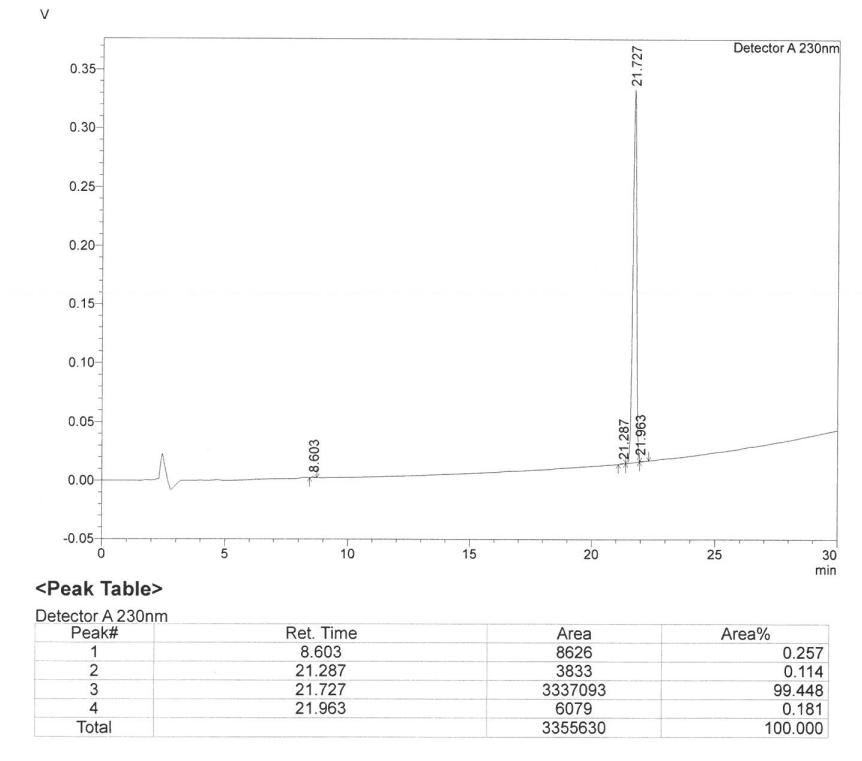
**

**Fig. S1**

**
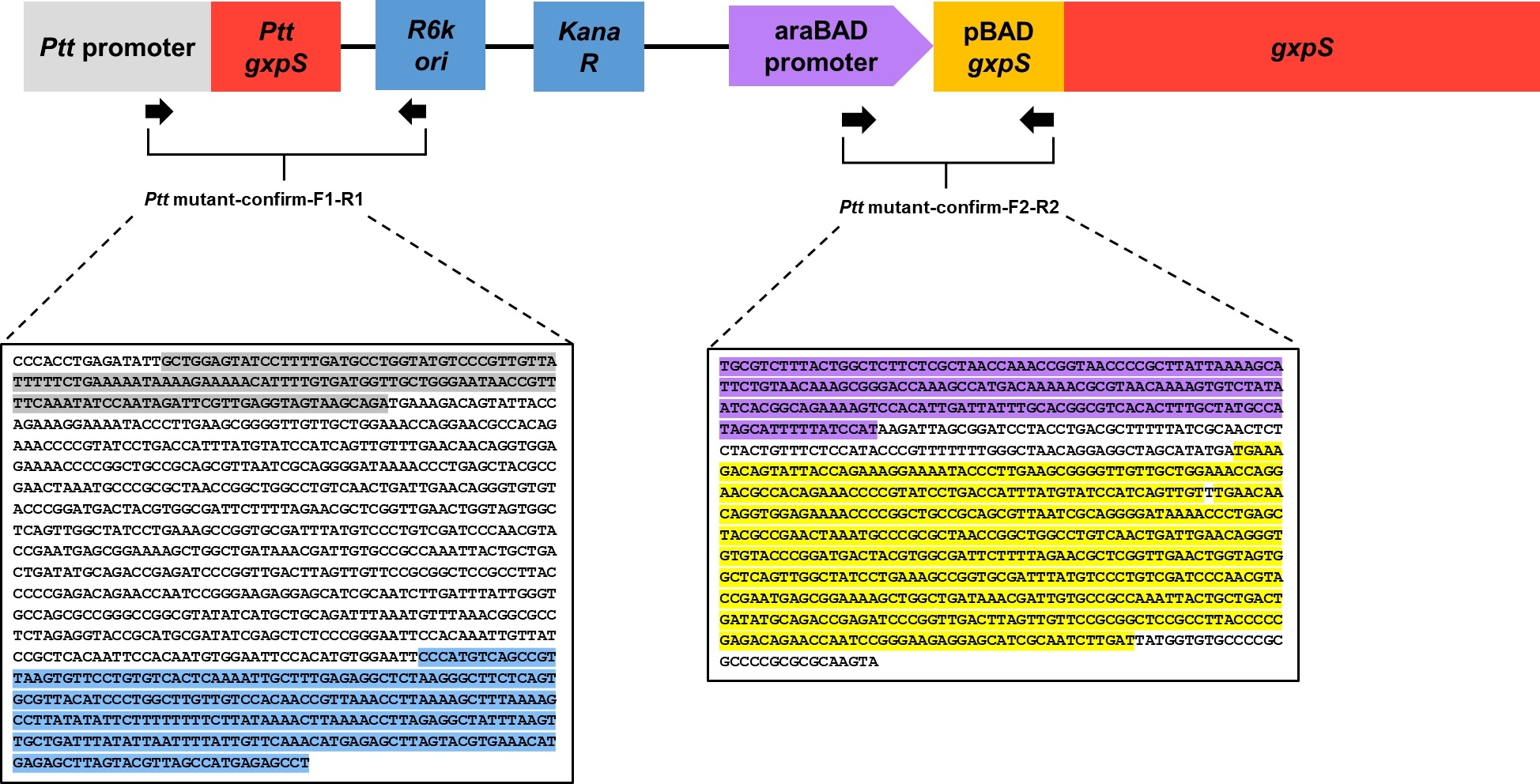
**

**Fig. S2**
